# Transforaminal posterior lumbar interbody fusion microscopic safe operating area: a three-dimensional model study based on computed tomography imaging

**DOI:** 10.1186/s13018-024-04830-9

**Published:** 2024-06-08

**Authors:** Wei Wang, Yukai Cui, Xiaohao Sun, Haoran Zhang, Wen Yin, Xilong Cui, Wei Jiao

**Affiliations:** 1 Department of Orthopaedic, Fuyang Hospital Affiliated with Bengbu Medical University (Fuyang People’s Hospital), Fuyang, China; 2 Department of Orthopaedic, Fuyang Hospital Affiliated with Anhui Medical University (Fuyang People’s Hospital), Fuyang, China; 3 School of Mechatronics Engineering and Automation, Shanghai University, 333 Nanchen Road, Shanghai, 200072 China; 4 Department of Orthopaedic, Anhui Provincial Clinical Medical Research Center for Spinal Deformities, Fuyang, China

**Keywords:** Endo-LIF, TPLIF, Kambin’s triangle, Secure region, Visible trephine, Three-dimensional reconstruction of lumbar spine CT

## Abstract

**Background:**

Endoscopic spine lumbar interbody fusion (Endo-LIF) is well-regarded within the academic community. However, it presents challenges such as intraoperative disorientation, high rates of nerve damage, a steep learning curve, and prolonged surgical times, often occurring during the creation of the operative channel. Furthermore, the undefined safe operational zones under endoscopy continue to pose risks to surgical safety. We aimed to analyse the anatomical data of Kambin’s triangle via CT imaging to define the parameters of the safe operating area for transforaminal posterior lumbar interbody fusion (TPLIF), providing crucial insights for clinical practice.

**Methods:**

We selected the L4–L5 intervertebral space. Using three-dimensional (3D), we identified Kambin’s triangle and the endocircle within it, and recorded the position of point ‘J’ on the adjacent facet joint as the centre ‘O’ of the circle shifts by angle ‘β.’ The diameter of the inscribed circle ‘d,’ the abduction angle ‘β,’ and the distances ‘L1’ and ‘L2’ were measured from the trephine’s edge to the exiting and traversing nerve roots, respectively.

**Results:**

Using a trephine with a diameter of 8 mm in TPLIF has a significant safety distance. The safe operating area under the TPLIF microscope was also clarified.

**Conclusions:**

Through CT imaging research, combined with 3D simulation, we identified the anatomical data of the L4–L5 segment Kambin’s triangle, to clarify the safe operation area under TPLIF. We propose a simple and easy positioning method and provide a novel surgical technique to establish working channels faster and reduce nerve damage rates. At the same time, according to this method, the Kambin’s triangle anatomical data of the patient’s lumbar spine diseased segments can be measured through CT 3D reconstruction of the lumbar spine, and individualised preoperative design can be conducted to select the appropriate specifications of visible trephine and supporting tools. This may effectively reduce the learning curve, shorten the time operation time, and improve surgical safety.

**Supplementary Information:**

The online version contains supplementary material available at 10.1186/s13018-024-04830-9.

## Background

The prevalence of degenerative lumbar diseases is increasing [1], particularly among younger individuals. Continuous advancements in minimally invasive lumbar spine techniques have positioned endoscopic spine lumbar interbody fusion (Endo-LIF) as a leading technology [2, 3], noted for its reduced surgical trauma and faster postoperative recovery than traditional open lumbar interbody fusion [4, 5]. However, Endo-LIF faces challenges including a steep learning curve [6, 7], possible disorientation during surgery [8], extended operation times [5, 9], and a risk of nerve damage [10, 11].

Said et al. [12] reported a 20% complication rate in Endo-TLIF patients, indicating that insufficient knowledge of safe operational areas during channel establishment could be contributing to these issues. Researchers are exploring further methods to enhance working channel efficiency [9], but many lack the anatomical data necessary to ensure surgical safety.

To overcome these limitations, this theoretical study focuses on the L4–L5 segment [13–15], often used for single-level lumbar fusion, and employs CT imaging to analyse Kambin’s triangle. We aimed to use imaging models to clarify the parameters of the safe operational area under transforaminal posterior lumbar interbody fusion (TPLIF), to provide valuable insights into the clinical application thereof.

## Methods

This study received approval from the Ethics Committee of the hospital in Fuyang, China (Permit No. [2022]33). Using the SYNGO system (SIEMENS, Germany), imaging data were retrospectively collected from patients who underwent three-dimensional (3D) lumbar spine CT reconstruction at the outpatient clinic from January 2022 to January 2023. Basic patient information, including name, sex, age, height, weight, and health status, was collected and the imaging data were screened according to specific criteria.

The images were obtained using a SOMATOM Definition AS (SIEMENS, Germany). CT scan sequences were exported to DICOM (.dcm) format and processed using Mimics software (Materialise, Version 21.0). A 3D spinal model of the L4–L5 lumbar segment was created, positioning the L4–L5 intervertebral space on the coronal, sagittal, and transverse planes. Anatomical measurements of Kambin’s triangle were recorded on the coronal and transverse planes. Two orthopaedic physicians at the hospital supervised the data collection process.

### Inclusion and exclusion criteria

We included patients undergoing 3D lumbar spine CT reconstruction at our hospital, aged 18 to 65, with clear CT imaging of the L4–L5 intervertebral space, and complete patient data. We excluded those with spinal scoliosis and kyphosis deformities; previous fractures, spondylolisthesis, or severe degenerative changes at the L4–L5 segment that narrow the intervertebral space; history of surgery, spinal tumours, tuberculosis, or infections at the L4–L5 segment; and incomplete patient data.

### Anatomic measurements

**Fig. 1:**
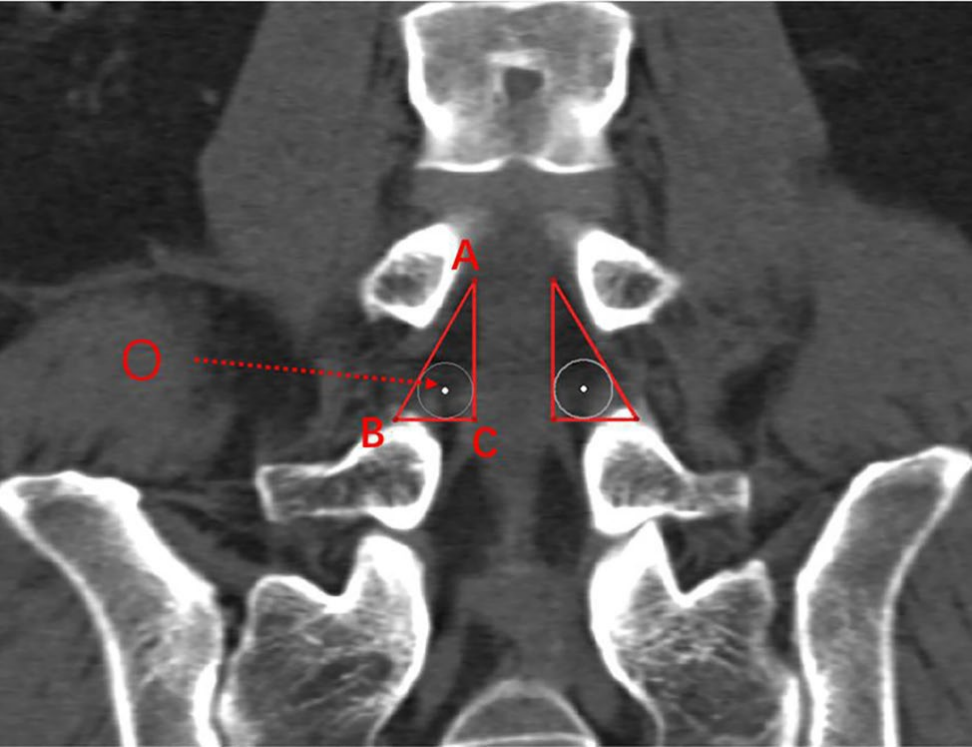
Kambin’s triangles on both sides of the L4–L5 intervertebral space. Images show the coronal plane, including the inscribed circles. AB, exiting nerve root; AC, traversing nerve root; BC, horizontal line at the upper edge of the pedicle; O, centre of the inscribed circle in Kambin’s triangle.Diameter of the inscribed circle in a right triangle = Base + Height − Hypotenuse (mm)

We identified and correctly position the L4–L5 intervertebral space in the coronal plane, and outlined the boundaries of “the working triangle” as suggested by Hardenbrook [16]. The hypotenuse of the triangle corresponds to the exiting nerve root, the base to the upper edge of the pedicle, and the height to the traversing nerve root. We then sketched Kambin’s triangle on the CT scan, and drew a circle within the triangle, labelling its centre as point ‘O,’ and measured the diameter, ‘d’ (in millimetres) of the inscribed circle. This diameter represents the maximum diameter of the visible trephine in the surgical field (Fig. 1).

**Fig. 2:**
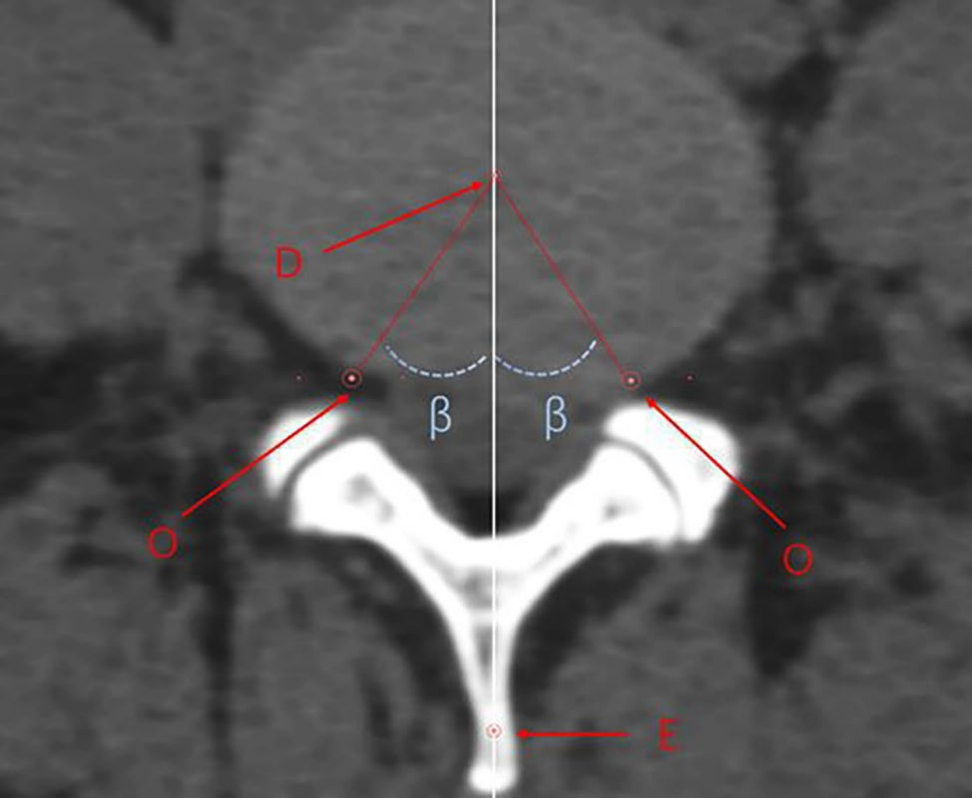
Coronal plane at the centre of the intervertebral disc within the L4–L5 space. D, centre of the intervertebral disc; O, centre of the inscribed circle in Kambin’s triangle; E, centre point of the spinous process; β, angle between O and D, which represents the abduction angle of the annular saw

On the sagittal plane, we adjusted the axis to ensure the horizontal baseline was parallel to the upper endplate of the L5 vertebra. We then elevated the horizontal baseline to align with the L4–L5 intervertebral space, thereby centring the plane on the intervertebral disc in the transverse section. A rectangle was then constructed by drawing tangents to the anterior, posterior, left, and right edges of the disc. The diagonals of the rectangle were connected to locate the centre of the intervertebral disc, labelled as point ‘D.’ From ‘D’, we traced backward to find the centre of the spinous process, defined as point ‘E,’ establishing DE as the central line. In this study, angle ‘β’ was measured between line OD, connecting centre ‘O’ with the centre of the intervertebral disc ‘D,’ and line DE, representing the trephine’s optimal abduction angle during surgery. Although centre ‘O’ is above the transverse section containing ‘D,’ the plane defined by lines DE and OD remains constant, ensuring accuracy of the measurement of angle ‘β’ on the transverse section (Fig. 2).

In the Mimics software 3D model, we identified the area where the centre ‘O,’ as projected by the angle ‘β,’ intersects with the facet joint on the same side. This intersection was designated as the focus point, represented by the letter ‘J.’ In the subsequent 3D model simulation of surgery, the area surrounding point ‘J’ served as the puncture fixation site for the Kirschner guide wire. Centred on the fixed Kirschner wire, a visible trephine was placed with a maximum diameter smaller than ‘d,’ ensuring it does not exceed the boundaries defined by ‘AB’ and ‘AC.’ Consequently, we identified the ‘J’ point as the safe central point during surgery, and the surrounding facet joint area as the safe zone (‘J’ point was showed in the supplementary material).

**Fig. 3:**
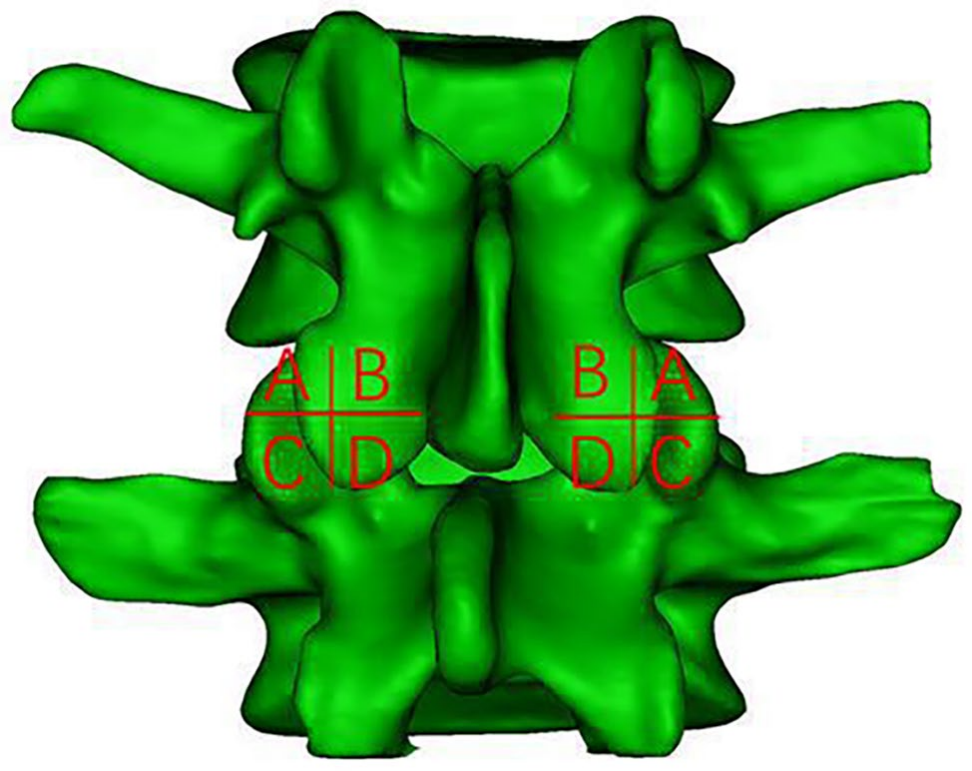
Mimics 3D simulation of the L4–L5 model from a posterior view. A, B, C, and D correspond to the upper outer, upper inner, lower outer, and lower inner quadrants of the divided facet joint, respectively

To precisely define the safe area around the ‘J’ point, we divided the facet joint into four equal quadrants using a 3D model in Mimics. The segmentation process included the following: (1) drawing a horizontal line at the upper vertex of the facet joint on the L5 vertebral body; (2) drawing a horizontal line at the lower edge of the facet joint under the L4 vertebral body; (3) drawing a vertical line from the lateral edge of the facet joint on the L5 vertebral body, perpendicular to the horizontal plane; (4) drawing a vertical line from the lateral edge of the exiting root, perpendicular to the horizontal plane. These steps produced four border lines by connecting the midpoints of lines 1 and 2, and lines 3 and 4. The resulting quadrants were labelled A, B, C, and D. The quadrant containing the ‘J’ point within the facet joint was specifically identified and recorded (Fig. 3).

**Fig. 4:**
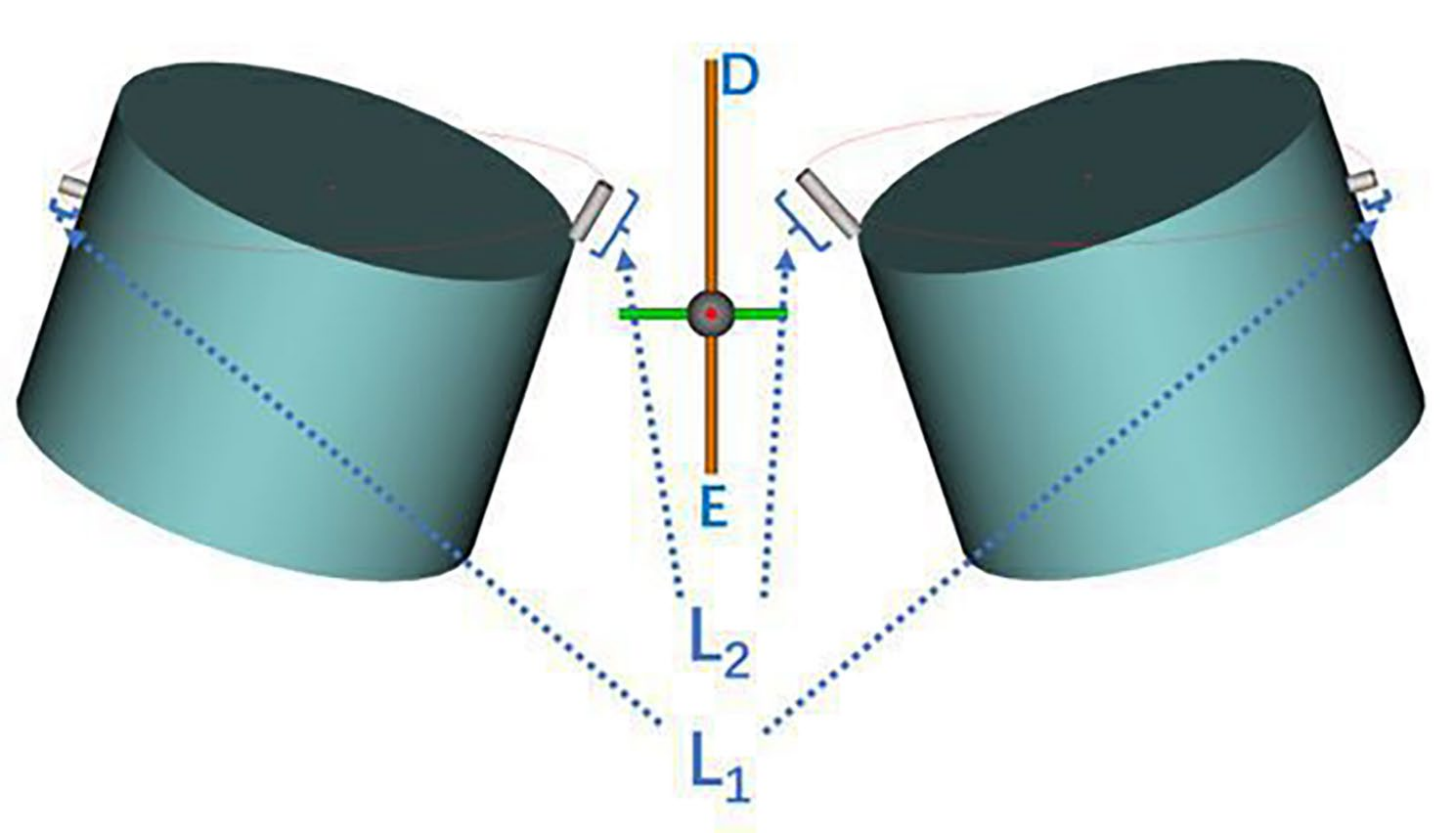
Mimics 3D simulation with an 8-mm diameter visible trephine. The trephine is placed on both sides of the intervertebral space. DE, midline, extending from the centre of the intervertebral disc on the transverse section to the midpoint of the spinous process; L1, shortest distance from the outer edge of the trephine to the side boundary of the exiting nerve root within Kambin’s triangle; L2, shortest distance from the outer edge of the trephine to the side boundary of the traversing nerve root within Kambin’s triangle

To enhance safety assessment, we compared the diameter ‘d’ to the commonly used 8 mm clinical diameter of a visible trephine. We then aimed to measure the shortest distance from the outer edge of the trephine, set at the ‘β’ abduction angle, to the lateral boundary of the exiting nerve root within Kambin’s triangle, denoted as ‘L1’; and the greatest distance to the lateral boundary of the traversing nerve root, denoted as ‘L2’. These measurements represent the safe distances of the visible trephine from the exiting and traversing nerve roots when establishing a working channel. We posited that results of ‘L1’ and ‘L2’ ≥0 mm indicate a sufficient safety margin of an 8-mm diameter visible trephine relative to both the exiting and traversing nerve roots; larger values suggest increased safety due to greater distances from these nerve roots. A result of ≤ 0 mm suggests that the outer edge of the visible trephine has reached or breached the exiting and traversing nerve roots, potentially causing damage during surgery (Fig. 4).

### Statistical analysis

Statistical analyses were conducted using SPSS version 26.0 (IBM, Armonk, NY). Quantitative data that were normally distributed are reported as mean ± standard deviation (SD), and those not normally distributed as median (interquartile range, IQR). Categorical data are presented as counts (percentage): n (%). Count data were analysed using the chi-square test. Depending on the distribution of the data, analyses were performed using either the one-sample t-test or the Wilcoxon rank-sum test, and the 95% confidence interval was calculated. A linear correlation analysis was employed to assess relationships between variables. *P* < 0.05 was considered statistically significant.

## Results

### Demographical characteristics and clinical data of the patients

Twenty patients were enrolled, comprising 8 males and 12 females. All patients underwent CT 3D reconstruction imaging of the lumbar L4–L5 segment. The demographic characteristics and clinical data of the enrolled patients are presented in Table 1.

**Table 1:** Demographic and clinical characteristics of the patients

Characteristic	Patients (*n* = 20)
Age (years)	48.45 ± 7.97
Sex, n (%)	
Male	8 (40%)
Female	12 (60%)
BMI, kg/m^2^	25.12 ± 2.13
Complication, n (%)	
Diabetes	1 (20%)
Hypertension	2 (10%)

BMI, body mass index

Age and BMI are expressed as mean ± SD. The remaining characteristics are expressed in terms of the number of patients (the percentage of the total number of patients)

### Measurements of Kambin’s triangle

The values for ‘d’ (mm), ‘L1,’ and ‘L2’ (mm) are shown in Table 2. In the dataset of 40 cases, the mean ‘β’ angle was 32.02 ± 0.90° (range, 30.32–34.56°).

The diameter ‘d’ of the inscribed circle averaged 11.62 mm (range, 11.35–12.04 mm), with extremes ranging from 9.46 mm to 12.54 mm. All measurements were significantly greater than the commonly used 8-mm visible trephine (*P* < 0.001, 95% confidence interval [CI]: 11.4–11.8°), and the average diameter was also significantly greater than 10 mm (*P* < 0.05, 95% CI: 11.4–11.8°).

The safety distance ‘L1’ averaged 1.40 mm (1.34–1.43 mm), ranging from 0.88 mm to 1.48 mm (95% CI: 1.3–1.4). ‘L2’ averaged 2.30 mm (2.14 to 2.34 mm), with a range from 1.99 mm to 3.43 mm (95% CI: 2.2–2.3). Both measurements were statistically significant (*P* < 0.001).

**Fig. 5:**
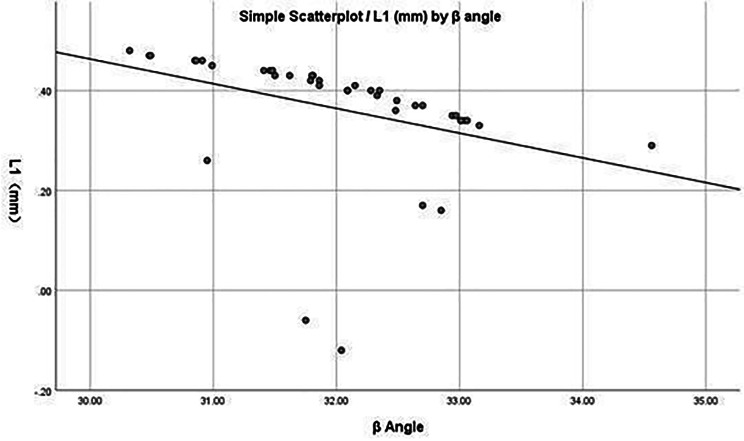
Linear correlation analysis between the β angle and L1. A negative correlation is observed, indicating that as the β angle increases, the safety distance L1 decreases

**Fig. 6:**
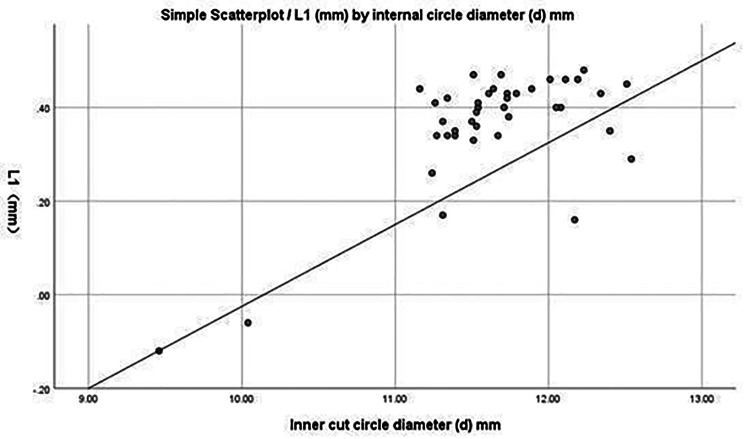
Linear correlation analysis between the diameter, d, of the inscribed circle and L1. A positive correlation is evident, showing that as the diameter increases, the safety distance, L1, also increases

The correlations between ‘β’ angle and ‘L1’ and between ‘d’ and ‘L1’ are presented in Figs. 5 and 6, respectively.

**Table 2:** Parameters of Kambin’s triangle

	Descriptive statistics				
	n	Median	Quartile	Minimum	Maximum
d (mm)	40	11.62	11.35,12.04	9.46	12.54
L1 (mm)	40	1.40	1.34,1.43	0.88	1.48
L2 (mm)	40	2.30	2.14,2.34	1.99	3.43

Values are expressed as median (quartile)

### Quadrant of the articular process joint where ‘J’ is located

An analysis of 40 cases demonstrated that the safety centre ‘J’ is consistently located in the upper external quadrant of the articular process joint, labelled as ‘A,’ with high statistical significance (*P* < 0.001). There were no statistically significant differences in the ‘β’ angle, diameter ‘d,’ distances ‘L1’ and ‘L2,’ and the location of ‘J’ with respect to patient sex, age, and BMI (*P* > 0.05). Similarly, no significant differences were found between the left and right sides of the L4–L5 intervertebral space (*P* > 0.05).

## Discussion

Using a trephine with a diameter of 8 mm in TPLIF has a significant safety distance. The safe operating area under the TPLIF microscope was also verified by our results, for intraoperative positioning reference and to propose a more efficient and safe working channel establishment method, providing an effective reference for clinical practice.

The primary challenge in Endo-LIF is the limited working space between the exiting and traversing nerve roots [6–8]. Currently, establishing the surgical endoscopic working channel requires the use of a radiofrequency ablator to remove the facet capsule and expose the articular processes [17, 18]. Additionally, this procedure involves using osteotomes, rongeurs, power drills, and trephines to resect part or all of the articular process bone before implantation of the working channel [19]. However, this procedure requires surgeons to possess an in-depth understanding of anatomical structures and the scope of the endoscopic operative area, thereby lengthening the learning curve for beginners.

The widespread adoption of visible trephines in endoscopic lumbar intervertebral fusion has prompted researchers to explore innovative methods to create working channels [9, 20]. For example, Kang Li et al. employed an 18-gauge percutaneous puncture at the upper facet joint space to guide the placement of the visible trephine. Subsequently, they used a 14-mm visible trephine to resect the facet joint and establish a working channel, which reduced surgery time and minimised nerve damage. This technique enhanced surgical visibility, facilitated haemostasis, and increased efficiency. However, positioning the 18-gauge needle and placing the trephine presents a challenge for beginners. Additionally, the use of large-diameter trephines has a lack of well-developed techniques and supporting anatomical data. Therefore, scholars emphasise the necessity of a thorough understanding of anatomical structures, knowledge of safe operational parameters, and the skill to establish percutaneous access to the intervertebral disc for successful Endo-LIF [10, 21, 22].

Safety is the most important consideration in research. In the study by Li [23], a method based on MRI lumbar nerve root water imaging to measure Kambin’s triangle anatomical data was reported, the PETLIF safe operating area was determined, and the concept of a “safety centre” was proposed. The authors pointed out that using the centre as the positioning point for operating sleeve insertion can remove the facet joint under blind vision and avoid nerve root damage as much as possible, which provides an effective reference for clinical practice. However, Li only measured Kambin’s triangle in the coronal plane; in this case, the operating sleeve is perpendicular to Kambin’s triangle on the coronal plane to remove bone and establish a working channel, but it is limited by the near-centre skin incision and the traversing nerve root, as well as the need for decompression range and intervertebral fusion cage implantation [24]. This vertical angle is only an ideal state and cannot be used in practice. Therefore, we added the measurement of the ‘β’ angle to simulate the abduction angle established by the working channel and, through 3D simulation, measured the safe distance represented by ‘L1’ and ‘L2’ under actual 3D imaging, and used this to evaluate the intraoperative work.

Regarding, the safety angle established by the channel, in the 40 cases of data, the ‘β’ angle was 32.02 ± 0.90°. In addition, a correlation analysis between the angle of abduction of the working channel and safety was included, showing a negative association between the ‘β’ angle and ‘L1,’ indicating that an increase in the abduction angle reduces the distance from the visible trephine to the exiting nerve root. However, no significant correlation was observed between the ‘β’ angle and ‘L2’ in this study.

In Endo-LIF, precise positioning can not only improve the efficiency of surgery, but also ensure the safety thereof. Li et al. [9] showed that the use of 18-gauge positioning mainly relies on fluoroscopy and surgeon experience; the requirements are high, and the selection of positioning points lacks anatomical data support. Although the “safe centre” point positioning method has corresponding anatomical data support, it is only described in the coronal plane [23]. The posterior bone mapped by the “safe centre” point was not included in the study by Li et al. [23]. During the operation, it was necessary to rely on the vertebral pedicle and sacrum as a reference for positioning points under fluoroscopy. This method was not easy to conduct. In a dataset of 40 cases, the authors demonstrated that, when the centre ‘O’ of the inscribed circle in Kambin’s triangle was abducted at the ‘β’ angle, it projected to the ‘J’ point. This point aligns with the initial Kirschner wire puncture location at the upper outer quadrant of the facet joint, showing high statistical significance (*P* < 0.001). The authors recommend inserting the initial Kirschner wire percutaneously into the upper outer quadrant of the facet joint under fluoroscopic guidance during surgery. After expanding the skin and soft tissue, the wire should be adjusted to an abduction angle of 32.02 ± 0.90° and aligned as parallel as possible to the intervertebral space before anchoring it within the facet joint bone. Using the Kirschner wire as a reference, the visible trephine is implanted and abducted at the same angle to guide bone resection and prevent disorientation during the procedure.

The measurement of Kambin’s triangle in this study found that the diameter ‘d’ of the inscribed circle was 11.62 mm (11.35, 12.04), with a maximum diameter of 12.54 mm and a minimum of 9.46 mm. That is, a visible trephine with a maximum diameter of 12.54 mm can pass through the operating area under the microscope, which is far greater that the diameter of 8 mm and 10 mm for visible trephines used clinically (*P <* 0.001, 95% CI: 11.4–11.8°). However, the minimum ‘d’ in the data is 9.46 mm; therefore, we believe that the intraoperative use of an 8-mm visible trephine is safer. The data obtained for ‘L1’ and ‘L2’ represent the safe distances of 1.40 mm (1.34, 1.43) and 2.30 mm (2.14, 2.34) (*P* < 0.001), respectively. That is, when an 8-mm visible trephine is implanted at 32.02 ± 0.90°, there will be no damage to the exiting and traversing nerve roots. We believe that this safe zone is significant. Furthermore, combined with the support of anatomical data, this effectively and significantly safely avoids the risk of nerve damage caused by the blind use of large-diameter visible trephines like those used by Li et al. [9].

**Fig. 7:**
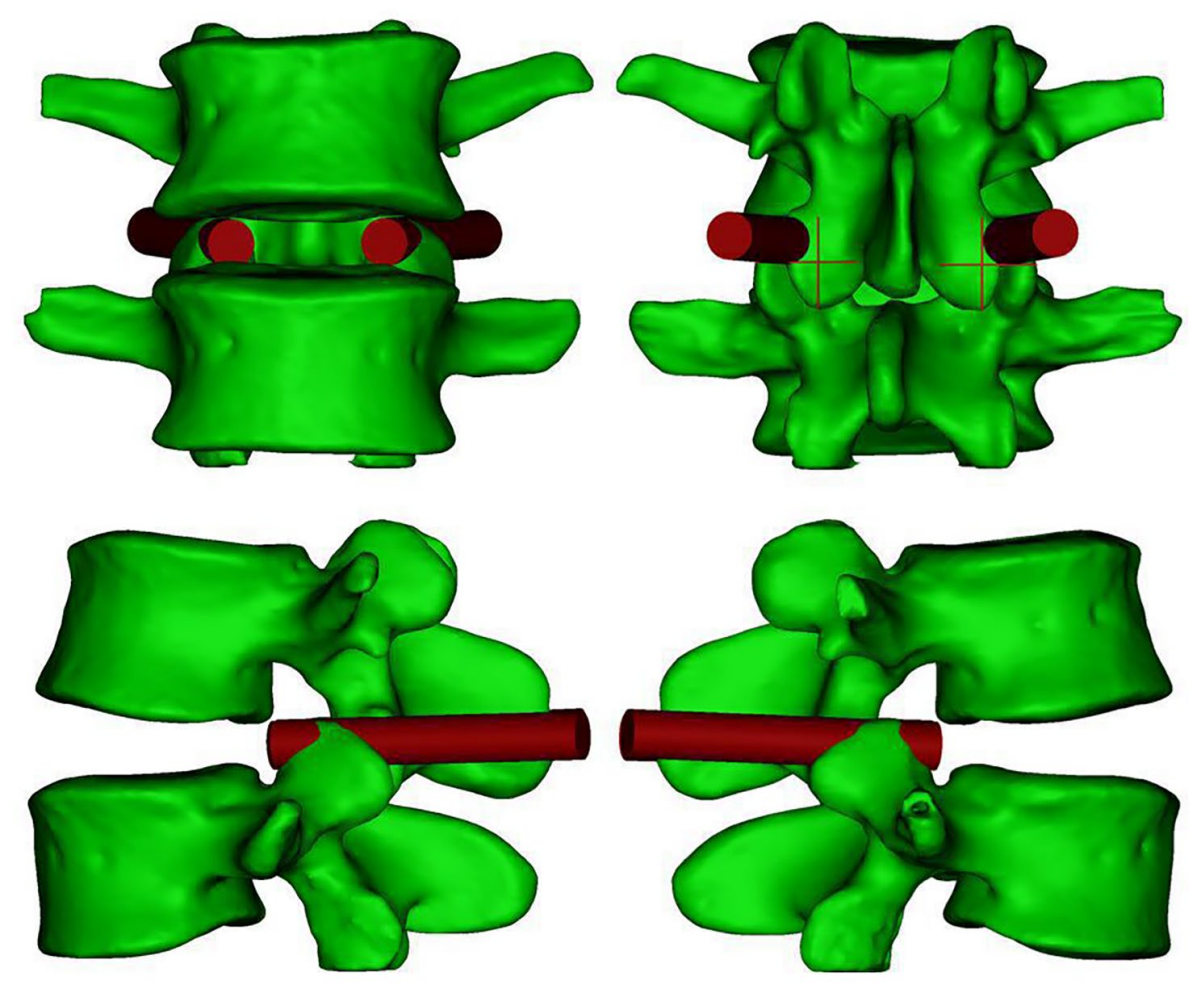
Mimics 3D simulation of the L4–L5 model. After the Kirschner wire is positioned in the upper outer quadrant of the facet joint, a visible trephine is centred on the wire to resect the facet joint and establish a working channel. The anteroposterior, anterior-posterior, left-lateral, and right-lateral views of a visible trephine abrasion of the articular synchondrosis to create a working channel are represented

Finally, we suggest utilising the data from this study in transforaminal lumbar interbody fusion (TLIF). During the procedure, a guide Kirschner wire is percutaneously inserted into the upper outer quadrant of the facet joint at the safety centre point ‘J,’ guided by C-arm fluoroscopy. The wire is then adjusted to an abduction angle of 32.02 ± 0.90° and aligned parallel to the intervertebral space before anchoring in the bone. Subsequently, the skin and soft tissue around the Kirschner wire are dilated, and an 8-mm visible trephine is inserted at the same abduction angle. This technique enables the resection of the facet joint bone in a single step, efficiently creating the working channel while ensuring a safe distance from the exiting and traversing nerve roots (Fig. 7).

### Limitations

This study also has some limitations. First, this study is a single-centre study with insufficient sample size. Further prospective multi-centre studies with large sample sizes are needed to provide more convincing results. Second, any impact on the L4–L5 section that is the normal size of Kambin’s triangle within the segment is excluded. Although this study has repeatedly confirmed the boundary position of Kambin’s triangle in the coronal plane, there may still be some errors. The Kambin’s triangle of healthy individuals may even be different due to anatomical differences. Finally, the safety distance in this study is within 3 mm, which still poses a great challenge. Therefore, the general applicability of this study to different patient groups needs to be further strengthened.

## Conclusion

Through CT imaging research, combined with 3D simulation, we studied the anatomical data of the L4–L5 segment Kambin’s triangle, which enabled clarification of the safe operation area under TPLIF. We not only propose a simple and easy positioning method, but also provide a novel surgical technique that establishes working channels faster and reduces nerve damage rates. At the same time, according to this method, the Kambin’s triangle anatomical data of the diseased segment of the patient’s lumbar spine can be measured through 3D reconstruction of lumbar spine CT, and preoperative individualised design can be conducted to select the appropriate specifications of the visible trephine and supporting tools. This can effectively reduce the learning curve, shorten the operation time, and improve surgical safety. Finally, this study is theoretical, and its results have been initially proven to be safe and feasible. In the future, we plan to conduct in vitro simulation research on 3D printing and apply our results to clinical practice.

### Electronic supplementary material

Below is the link to the electronic supplementary material.

Supplementary Material 1

## Data Availability

No datasets were generated or analysed during the current study.

## Abbreviations

Endo-LIF: Endoscopic spine lumbar interbody fusion
TLIF: Transforaminal lumbar interbody fusion
TPLIF: Transforaminal posterior lumbar interbody fusion
SD: Standard deviation
