# Supplementary material for: Transforaminal posterior lumbar interbody fusion microscopic safe operating area: a three-dimensional model study based on computed tomography imaging
Source: J Orthop Surg Res. 2024 Jun 8;19:342. doi: 10.1186/s13018-024-04830-9 (PMC11161984; doi:10.1186/s13018-024-04830-9)
Supplement: Supplementary file 1 — Supplementary Material 1 [file 13018_2024_4830_MOESM1_ESM.docx]

**Additional pictures and descriptions**

In the Mimics software 3D model, we identified the area where the centre ‘O,’ as projected by the angle ‘β,’ intersects with the facet joint on the same side. This intersection was designated as the focus point, represented by the letter ‘J.’ In the subsequent 3D model simulation of surgery, the area surrounding point ‘J’ served as the puncture fixation site for the Kirschner guide wire. Centred on the fixed Kirschner wire, a visible trephine was placed with a maximum diameter smaller than ‘d,’ ensuring it does not exceed the boundaries defined by ‘AB’ and ‘AC.’ Consequently, we identified the ‘J’ point as the safe central point during surgery, and the surrounding facet joint area as the safe zone.

**
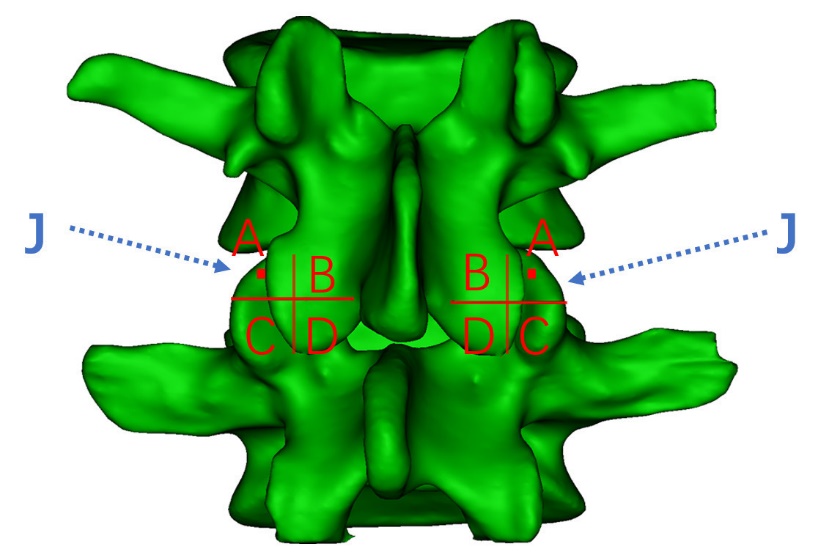
**
